# Mental health first aid responses of the public: results from an Australian national survey

**DOI:** 10.1186/1471-244X-5-9

**Published:** 2005-02-06

**Authors:** Anthony F Jorm, Kelly A Blewitt, Kathleen M Griffiths, Betty A Kitchener, Ruth A Parslow

**Affiliations:** 1 Centre for Mental Health Research, Australian National University, Canberra, ACT 0200, Australia

## Abstract

**Background:**

The prevalence of mental disorders is so high that members of the public will commonly have contact with someone affected. How they respond to that person (the mental health first aid response) may affect outcomes. However, there is no information on what members of the public might do in such circumstances.

**Methods:**

In a national survey of 3998 Australian adults, respondents were presented with one of four case vignettes and asked what they would do if that person was someone they had known for a long time and cared about. There were four types of vignette: depression, depression with suicidal thoughts, early schizophrenia, and chronic schizophrenia. Verbatim responses to the open-ended question were coded into categories.

**Results:**

The most common responses to all vignettes were to encourage professional help-seeking and to listen to and support the person. However, a significant minority did not give these responses. Much less common responses were to assess the problem or risk of harm, to give or seek information, to encourage self-help, or to support the family. Few respondents mentioned contacting a professional on the person's behalf or accompanying them to a professional. First aid responses were generally more appropriate in women, those with less stigmatizing attitudes, and those who correctly identified the disorder in the vignette.

**Conclusions:**

There is room for improving the range of mental health first aid responses in the community. Lack of knowledge of mental disorders and stigmatizing attitudes are important barriers to effective first aid.

## Background

Surveys in many countries have found that mental disorders have a high prevalence and are a major cause of disability in the population [1-3]. For example, the Australian National Survey of Mental Health and Wellbeing found that close to one in five adults met the criteria for a mental disorder at some time during the 12 months before the survey [3]. The most common mental disorders were anxiety (10%), depressive (6%) and substance use disorders (8%). These disorders are so prevalent that everyone in the community can expect to have close contact with someone experiencing a mental disorder.

How people initially respond to others with a mental disorder may influence their recovery. For example, it is known that many people with mental disorders get no professional help [4] and that help-seeking is more likely if relatives or friends suggest it [5]. It is also known that recovery can be assisted if family members are supportive and not critical [6]. This type of initial help from a person's social network can be defined as mental health first aid.

There has been no previous research on mental health first aid knowledge in the population. Previous mental health literacy surveys have assessed knowledge and beliefs about mental disorders and their treatment [5], but these surveys have not assessed the public's intentions for mental health first aid responses to hypothetical cases of persons with mental disorders. Thus it is not known which mental health first aid responses are currently adequate and which need improving. Hence a relevant question was asked in a recent national survey of mental health literacy in Australia.

## Methods

### The Australian survey

In 2003–2004 a household survey was carried out of Australian adults aged 18 or over by the company AC Nielsen. Households were sampled from 250 census districts covering all states and territories and metropolitan and rural areas. Up to 5 call backs were made to metropolitan selections and 3 to non-metropolitan selections. To achieve a target sample of 4,000 interviews with adults aged 18 years or over, visits were made to 28,947 households. The outcome of these visits was: no contact after repeated visits 14,630; vacant house or lot 306; refused 7,815; person sampled within household temporarily unavailable 1,132; no suitable respondent in household 287; did not speak English 383; incapable of responding 213; and unavailable for the duration of the survey 181. The achieved sample was 3998 persons, with 1001 receiving the depression vignette, 999 the depression with suicidal thoughts vignette, 997 the early schizophrenia vignette, and 1001 the chronic schizophrenia vignette.

### Interview content

The interview was based on a vignette of a person with a mental disorder. On a random basis, respondents were shown one of four vignettes: a person with major depression, one with major depression together with suicidal thoughts, a person with early schizophrenia, and one with chronic schizophrenia. All vignettes were written to satisfy the diagnostic criteria for either major depression or schizophrenia according to DSM-IV and ICD-10. The vignette with depression and the one with early schizophrenia were written to satisfy these diagnostic criteria at a minimal level, so that we could ascertain the public's reaction to cases of a developing disorder which had reached the point where intervention was needed. The vignette of the person with depression together with suicidal thoughts was identical to the depression vignette in all respects except the suicidal thoughts and was designed to assess how this symptom affected the public's response. The chronic schizophrenia vignette was designed to assess the response to someone with a severe long-standing disorder, where acceptance seemed less likely. Respondents were also randomly assigned to receive either male ("John") or female ("Mary") versions of the vignette. These vignettes (John version) are shown in Table 1.

**Table 1:** Case vignettes used in the survey

**Disorder**	**Vignette**
Depression	John is 30 years old. He has been feeling unusually sad and miserable for the last few weeks. Even though he is tired all the time, he has trouble sleeping nearly every night. John doesn't feel like eating and has lost weight. He can't keep his mind on his work and puts off making decisions. Even day-to-day tasks seem too much for him. This has come to the attention of his boss, who is concerned about John's lowered productivity.
Depression with suicidal thoughts	John is 30 years old. He has been feeling unusually sad and miserable for the last few weeks. Even though he is tired all the time, he has trouble sleeping nearly every night. John doesn't feel like eating and has lost weight. He can't keep his mind on his work and puts off making any decisions. Even day-to-day tasks seem too much for him. This has come to the attention of John's boss who is concerned about his lowered productivity. John feels he will never be happy again and believes his family would be better off without him. John has been so desperate, he has been thinking of ways to end his life.
Early schizophrenia	John is 24 and lives at home with his parents. He has had a few temporary jobs since finishing school but is now unemployed. Over the last six months he has stopped seeing his friends and has begun locking himself in his bedroom and refusing to eat with the family or to have a bath. His parents also hear him walking about his bedroom at night while they are in bed. Even though they know he is alone, they have heard him shouting and arguing as if someone else is there. When they try to encourage him to do more things, he whispers that he won't leave home because he is being spied upon by the neighbour. They realize he is not taking drugs because he never sees anyone or goes anywhere.
Chronic schizophrenia	John is 44 years old. He is living in a boarding house in an industrial area. He has not worked for years. He wears the same clothes in all weathers and has left his hair to grow long and untidy. He is always on his own and is often seen sitting in the park talking to himself. At times he stands and moves his hands as if to communicate to someone in nearby trees. He rarely drinks alcohol. He speaks carefully using uncommon and sometimes made-up words. He is polite but avoids talking with other people. At times he accuses shopkeepers of giving information about him to other people. He has asked his landlord to put extra locks on his door and to remove the television set from his room. He says spies are trying to keep him under observation because he has secret information about international computer systems which control people through television transmitters. His landlord complains that he will not let him clean the room which is increasingly dirty and filled with glass objects. John says he is using these "to receive messages from space".

After being presented with the vignette, respondents were asked a series of questions to assess their recognition of the disorder in the vignette, their beliefs about treatment and long-term outcomes, beliefs about causes and risk factors, stigmatizing attitudes, awareness of mental disorders in the media, contact with people like those in the vignette, and the health and sociodemographic characteristics of the respondent. The questions relevant to the present paper are described below.

To assess recognition of the problem in the vignette, respondents were asked: "What would you say, if anything, is wrong with John?". Responses of "depression" were counted as correct for the first two vignettes above, and responses of "schizophrenia" or "psychosis" for the second two. To assess mental health first aid responses, respondents were asked the open-ended question: "Imagine John is someone you have known for a long time and care about. You want to help him. What would you do?". Answers were recorded verbatim by the interviewer. To assess contact with people like those in the vignette, respondents were asked: "Has anyone in your family or close circle of friends ever had problems similar to John's?"; "Have they received any professional help or treatment for these problems?'; "Have you ever had problems similar to John's?"; "Have you received any professional help or treatment for these problems?"; and "Have you ever had a job that involved providing treatment or services to a person with a problem like John's?". Those that said "yes" to these questions were respectively labelled in the analyses reported below as "carers", "consumers" or "professionals". To assess stigma, respondents were asked a series of nine questions designed to elicit their attitudes towards the person in the vignette (personal stigma) and nine items concerning what they thought others in the community would believe about the person in the vignette (perceived stigma) [7]. Personal stigma items were of the form: "Please indicate how strongly you agree or disagree with each statement. People with a problem like John's could snap out of it if they wanted. Strongly agree, Agree, Neither agree nor disagree, Disagree, Strongly disagree". Perceived stigma items were of the form: "Now we would like you to tell us what you think most other people believe. Please indicate how strongly you agree or disagree with the following statements. Most people believe that people with a problem like John's could snap out of it if they wanted. Strongly agree, Agree, Neither agree nor disagree, Disagree, Strongly disagree". Sociodemographic characteristics recorded included age group (coded as 18–39, 40–59 and 60+ years), gender, and education (dichotomized as bachelor's degree or higher versus lower-level qualifications).

### Content analysis of responses to open-ended question

Responses were coded according to the categories identified from an earlier study where the same question was administered as part of a randomized controlled trial of Mental Health First Aid [8]. Responses were coded with a "yes" or "no" in each category, such that multiple categories were possible. The categories were:

A. Encourage professional help-seeking

B. Listen to / talk to / support person

C. Listen to / talk to / support family

D. Assess problem / assess risk of harm

E. Give or seek information

F. Encourage self-help

Responses coded into category *A. Encourage professional help-seeking*, were subcoded into multiple categories to identify the type of professional help recommended. These categories were:

A1. GP / doctor unspecified

A2. Counsellor

A3. Psychiatrist

A4. Psychologist

A5. Mental health team / services

A6. Other mental health professionals

A7. Unspecified professionals and other professionals

A8. Accompany person (eg. Offer to go with him/her)

A9. Contact help on their behalf

Examples of responses coded into category B included "support, understanding and caring, someone to talk to him", "talk to her about it", "listen", "be there for him".

Responses coded into category C were the same for category B, but referred to giving support, listening to, or talking to the sufferer's family. For example, "talk to her family", "contact relatives", "ask advice of parents", "support his parents".

Responses coded into category D included "keep an eye on her, make sure she is safe", "make a contract with her so if she wants to harm herself she rings me first", "find out what is the real problem behind the behaviour, focus on the problem".

Responses coded into category E included "ring the local health authority to get advice", "talk to other people who have been in the situation", "speak to health professionals and get best advice", "get some brochures from community health and give them to him". Responses coded into category F included "I suggest that he have a holiday/exercise/change jobs", "try and help him get into something he is interested in", "support groups", "do something for herself to get out of the situation".

Inter-rater reliability of the coding was assessed by a second rater who independently coded 100 responses which were randomly selected using the SPSS Select Cases procedure [9].

### Statistical analysis

Inter-rater reliability of the content coding was assessed using kappa. Kappa values were interpreted according to Altman [10] as follows: 0.8–1.0 very good; 0.6–0.8 good; 0.4–0.6 moderate; 0.2–0.4 fair; and <0.2 poor.

The frequency of codings was analysed by pooling across male and female versions of each vignette and percent frequencies calculated. Percentages were calculated applying survey weights to give better population estimates. Standard errors of these percentages were estimated using the Complex Samples procedure in SPSS 12.0 [9]. This procedure takes account of sampling weights and geographic clustering in the sample. For simplicity of exposition, the standard errors are not reported for each estimate. However, they were always <2%. Such a standard error implies that a difference of 4% between vignettes was always statistically significant at the P < 0.05 level.

Multiple logistic regressions were then conducted to examine the levels of association between participants suggesting particular treatment options and their sociodemographic and mental health experience attributes. The following predictor variables were included: age group; consumer, carer and professional status, including whether or not professional help was obtained; and levels of perceived stigma and personal stigma. Two vignette measures – type of vignette provided and whether respondents correctly identified the problem portrayed in that vignette – were also included in the analyses. Each logistic regression was also adjusted to take into account of sampling weights and clustering method applied in this survey. These analyses were undertaken using STATA 8 [11].

## Results

### Reliability of coding open-ended responses

Inter-rater reliability was assessed for a randomly chosen 100 responses. Kappa was very good or good for encourage professional help-seeking (0.89), listen/talk/support person (0.70), listen/talk/support family (1.00), encourage seeing doctor (0.98), encourage seeing counsellor (0.93), encourage seeing psychiatrist (0.94), encourage seeing psychologist (0.88), and accompanying the person to a professional (0.95). It was moderate for give or seek information (0.48), encourage seeing unspecified and other professionals (0.56), and contact professional on their behalf (0.56). Kappa was fair for encourage self-help (0.34) and poor for assess problem/risk of harm (0.15). Kappa could not be calculated because of zero frequencies from the first rater for the categories of mental health team/services and other mental health professional.

To better understand the reasons for the fair and poor agreement with two of the codes, positive and negative agreement were examined separately [12]. In both cases, negative agreement was high (0.95 for both), but positive agreement was low (0.38 and 0.15 respectively). The low positive agreement resulted because the second rater used these codes much less frequently than the first rater. However, despite this difference, neither rater used these two codes frequently, suggesting a low frequency of these responses in the population.

### Frequencies of mental health first aid responses

Table 2 shows the percentage frequency of each category of response. The most common responses for all vignettes were to encourage professional help-seeking and to listen/talk/support the person. The differences between the vignettes were comparatively small. For the chronic schizophrenia vignette, there was a greater frequency of encouraging professional help-seeking and giving or seeking information, and a lesser frequency of listen/talk/support the person. Assessing the problem/risk of harm was more common for the depression vignettes than for the schizophrenia vignettes. Listen/talk/support family was more common for the early schizophrenia vignette, which was the only one to specifically mention family members.

**Table 2:** Percentage of respondents who mentioned various first aid responses

**Response**	**Depression Vignette**	**Depression/Suicidal Vignette**	**Early Schizophrenia Vignette**	**Chronic Schizophrenia Vignette**
Encourage professional help-seeking	58.6	62.4	57.5	66.0
Listen/ talk/ support person	69.1	73.4	70.3	65.6
Listen/ talk/ support family	2.2	2.8	8.5	2.4
Assess problem/ risk of harm	17.3	14.8	9.7	6.8
Give or seek information	5.5	7.1	9.1	12.9
Encourage self-help	12.0	10.8	12.3	10.2

Table 3 shows types of professionals mentioned by respondents who encouraged professional help-seeking. The most commonly mentioned was GP/doctor unspecified. This recommendation was more common for the depression vignettes than for the schizophrenia vignettes. Conversely, psychiatrists were mentioned more for the schizophrenia vignettes.

**Table 3:** Percentage of respondents who mentioned encouraging help-seeking with various types of professionals

**Type of Professional**	**Depression Vignette**	**Depression/ Suicidal Vignette**	**Early Schizophrenia Vignette**	**Chronic Schizophrenia Vignette**
GP/ doctor unspecified	40.1	35.9	26.9	27.4
Counsellor	8.1	8.3	7.6	7.3
Psychiatrist	2.9	3.3	6.4	7.3
Psychologist	2.2	2.5	2.5	3.1
Mental health team/services	0.1	0.4	1.5	2.3
Other mental health professional	0.2	0.2	1.0	1.1
Unspecified professionals or other professionals	14.1	21.1	20.9	29.9

Table 4 gives the percentage frequency of ways of encouraging professional help-seeking. Accompanying the person was more common for the chronic schizophrenia vignette, while contacting the professional on the person's behalf was more common for both the schizophrenia vignettes.

**Table 4:** Percentage of respondents who mentioned ways of encouraging professional help-seeking

**Type of Professional**	**Depression Vignette**	**Depression/ Suicidal Vignette**	**Early Schizophrenia Vignette**	**Chronic Schizophrenia Vignette**
Accompany person	8.7	11.0	9.7	16.7
Contact professional on their behalf	3.0	4.1	12.8	15.9

### Predictors of responses

Table 5 shows the predictors of first aid responses from the multiple logistic regressions. Taking predictors with P <0.01, encouraging professional help-seeking was more likely in response to the chronic schizophrenia vignette, from women, and from those who correctly recognized the problem in the vignette. It was less likely from consumers who had not sought help and respondents high on personal stigma. Listening/talking/supporting the person was more likely from consumers who had sought help. Listening/talking/supporting the family was more likely in response to the chronic schizophrenia vignette, and less likely from those high on personal stigma. Assessing the problem/ risk of harm was less likely in response to either schizophrenia vignette and from people aged 60+. Giving or seeking information was more likely in response to either schizophrenia vignette and from those who perceived stigma in others, but less likely from those high in personal stigma. Encouraging self-help was more likely from respondents high in personal stigma and less likely from those who correctly recognized the problem in the vignette.

**Table 5:** Odds ratios (and P-values) from multiple logistic regression analyses predicting first aid responses

**Predictor**	**Encourage professional help-seeking**	**Listen/ talk/ support person**	**Listen /talk/ support family**	**Assess problem/ risk of harm**	**Give or seek information**	**Encourage self-help**
**Type of vignette**						
Depression	1.00^1^	1.00^1^	1.00^1^	1.00^1^	1.00^1^	1.00^1^
Depression/ suicidal	1.13 (0.284)	1.19 (0.149)	1.28 (0.433)	0.82 (0.154)	1.21 (0.360)	0.87 (0.385)
Early schizophrenia	0.95 (0.636)	1.13 (0.298)	4.67 (0.000)	0.46 (0.000)	1.83 (0.002)	1.07 (0.671)
Chronic schizophrenia	1.66 (0.000)	0.88 (0.266)	1.00 (0.997)	0.32 (0.000)	2.83 (0.000)	0.75 (0.100)
**Sociodemographic characteristics**						
Age 18–39	1.00^1^	1.00^1^	1.00^1^	1.00^1^	1.00^1^	1.00^1^
Age 40–59	1.04 (0.646)	0.89 (0.138)	0.91 (0.645)	0.94 (0.628)	0.87 (0.341)	1.04 (0.759)
Age 60+	0.97 (0.753)	0.79 (0.010)	0.85 (0.515)	0.64 (0.002)	0.66 (0.031)	1.04 (0.809)
Female gender	1.32 (0.000)	1.20 (0.024)	0.92 (0.663)	0.74 (0.015)	0.99 (0.937)	0.87 (0.247)
University degree	1.20 (0.065)	1.07 (0.428)	0.92 (0.660)	1.19 (0.218)	1.01 (0.934)	1.06 (0.676)
**Experience with mental disorders**						
Consumer – not sought help	0.50 (0.000)	1.47 (0.061)	1.56 (0.291)	1.54 (0.047)	0.61 (0.212)	1.77 (0.010)
Consumer – sought help	0.88 (0.314)	1.49 (0.000)	0.47 (0.024)	1.02 (0.920)	0.78 (0.247)	1.12 (0.514)
Carer – not sought help	0.78 (0.149)	1.08 (0.666)	0.48 (0.195)	1.11 (0.658)	1.01 (0.965)	1.17 (0.503)
Carer – sought help	1.22 (0.032)	1.02 (0.841)	1.09 (0.668)	0.83 (0.187)	1.05 (0.741)	0.98 (0.900)
Professional	0.92 (0.403)	1.20 (0.068)	1.19 (0.400)	0.97 (0.853)	0.96 (0.801)	1.06 (0.679)
**Stigma**						
Personal stigma	0.95 (0.000)	0.99 (0.494)	0.93 (0.001)	1.01 (0.612)	0.95 (0.000)	1.06 (0.000)
Perceived stigma	1.01 (0.112)	0.99 (0.300)	1.00 (0.883)	1.00 (0.876)	1.04 (0.003)	0.99 (0.580)
Correct recognition of disorder in vignette	1.60 (0.000)	1.05 (0.550)	1.22 (0.320)	1.27 (0.095)	1.32 (0.060)	0.70 (0.007)

^1^Reference category

Table 6 shows predictors of encouraging help-seeking with various types of health professionals. Encouraging the person to see a GP or doctor was more likely in response to either schizophrenia vignette, from women and from those who correctly recognized the problem in the vignette, while it was less likely in those high on personal stigma. Encouraging help-seeking from a counsellor was less likely from those aged 60+. Encouraging help-seeking from a psychiatrist was more likely in response to either schizophrenia vignette, while encouraging help-seeking from a psychologist was more likely from the university educated. Encouraging help-seeking from a mental health team/services was more likely in response to the chronic schizophrenia vignette and from those with professional experience in the area of mental health. Encouraging help-seeking from other mental health professionals was less likely in respondents high on personal stigma, while encouraging it from unspecified professionals was more likely in response to the depression/suicidal vignette, to either schizophrenia vignette, and from respondents who perceive stigma in others, while it was less likely in those high on personal stigma.

**Table 6:** Odds ratios (and P-values) from multiple logistic regression analyses predicting encouragement of help-seeking from various types of professionals

**Predictor**	**GP/ doctor unspecified**	**Counsellor**	**Psychiatrist**	**Psychologist**	**Mental health team/ services**	**Other mental health professionals**	**Unspecified professionals or other professionals**
**Type of vignette**							
Depression	1.00^1^	1.00^1^	1.00^1^	1.00^1^	1.00^1^	1.00^1^	1.00^1^
Depression/ suicidal	0.77 (0.012)	1.05 (0.797)	1.04 (0.895)	1.13 (0.688)	1.92 (0.597)	1.28 (0.784)	1.76 (0.000)
Early schizophrenia	0.55 (0.000)	0.92 (0.697)	2.23 (0.002)	1.42 (0.281)	13.79 (0.013)	3.68 (0.102)	1.72 (0.000)
Chronic schizophrenia	0.60 (0.000)	1.16 (0.461)	2.66 (0.000)	1.76 (0.062)	20.34 (0.004)	7.12 (0.024)	2.96 (0.000)
**Sociodemographic characteristics**							
Age 18–39	1.00^1^	1.00^1^	1.00^1^	1.00^1^	1.00^1^	1.00^1^	1.00^1^
Age 40–59	1.19 (0.055)	0.88 (0.366)	1.35 (0.096)	0.96 (0.887)	0.50 (0.112)	1.03 (0.949)	0.93 (0.476)
Age 60+	1.35 (0.010)	0.49 (0.001)	1.55 (0.044)	1.24 (0.505)	1.00 (1.00)	0.54 (0.447)	0.74 (0.017)
Female gender	1.34 (0.000)	1.29 (0.08)	0.74 (0.061)	1.06 (0.823)	1.00 (1.00)	1.22 (0.676)	1.10 (0.294)
University degree	0.96 (0.638)	1.34 (0.054)	0.87 (0.473)	2.29 (0.001)	1.80 (0.132)	1.89 (0.148)	1.12 (0.264)
**Experience with mental disorders**							
Consumer – not sought help	0.69 (0.074)	0.63 (0.237)	0.38 (0.126)	0.86 (0.813)	-^2^	-^2^	0.61 (0.056)
Consumer – sought help	0.91 (0.365)	1.23 (0.227)	0.82 (0.457)	1.64 (0.060)	1.02 (0.967)	1.22 (0.735)	0.88 (0.334)
Carer – not sought help	0.87 (0.477)	1.10 (0.759)	1.53 (0.255)	0.96 (0.938)	0.88 (0.907)	0.78 (0.817)	0.97 (0.904)
Carer – sought help	1.08 (0.389)	1.32 (0.074)	1.46 (0.025)	1.51 (0.110)	1.05 (0.908)	1.08 (0.842)	1.01 (0.916)
Professional	1.00 (0.985)	0.95 (0.751)	0.891 (0.570)	1.17 (0.570)	2.42 (0.008)	1.01 (0.990)	0.98 (0.828)
**Stigma**							
Personal stigma	0.97 (0.005)	1.00 (0.969)	1.00 (0.835)	1.00 (0.917)	0.92 (0.120)	0.89 (0.007)	0.95 (0.000)
Perceived stigma	0.99 (0.079)	1.01 (0.468)	1.00 (0.969)	0.98 (0.439)	1.04 (0.188)	1.05 (0.083)	1.03 (0.000)
Correct recognition of disorder in vignette	1.43 (0.000)	1.49 (0.019)	1.17 (0.379)	0.74 (0.171)	1.60 (0.255)	3.00 (0.072)	1.24 (0.031)

^1^Reference category

^2^Could not be calculated

Table 7 shows predictors of ways of encouraging professional help-seeking. Accompanying the person to professional help was more likely in response to the chronic schizophrenia vignette and from women. Contacting the professional on the person's behalf was more likely in response to either schizophrenia vignette and from respondents who perceived stigma in others, while it was less likely from those high on personal stigma.

**Table 7:** Odds ratios (and P-values) from multiple logistic regression analyses predicting ways of encouraging professional help-seeking

**Predictor**	**Accompany person**	**Contact professional on their behalf**
**Type of vignette**		
Depression	1.00^1^	1.00^1^
Depression/ suicidal	1.21 (0.285)	1.68 (0.071)
Early schizophrenia	1.08 (0.646)	5.27 (0.000)
Chronic schizophrenia	2.26 (0.000)	8.01 (0.000)
**Sociodemographic characteristics**		
Age 18–39	1.00^1^	1.00^1^
Age 40–59	1.07 (0.549)	0.97 (0.828)
Age 60+	0.90 (0.485)	0.63 (0.017)
Female gender	1.43 (0.003)	1.06 (0.683)
University degree	0.86 (0.220)	1.31 (0.046)
**Experience with mental disorders**		
Consumer – not sought help	0.51 (0.060)	0.33 (0.068)
Consumer – sought help	1.19 (0.228)	0.71 (0.136)
Carer – not sought help	0.83 (0.491)	1.16 (0.620)
Carer – sought help	0.98 (0.893)	1.12 (0.424)
Professional	1.21 (0.109)	1.12 (0.427)
**Stigma**		
Personal stigma	0.99 (0.659)	0.95 (0.000)
Perceived stigma	0.99 (0.283)	1.03 (0.008)
Correct recognition of disorder in vignette	1.16 (0.280)	1.37 (0.028)

^1^Reference category

## Discussion

The most common first aid responses were found to be encouraging professional help-seeking and listening/talking/supporting the person. Nevertheless, these responses were far from universal, with 32–44% not mentioning professional help and 27–34% not mentioning listening/talking/supporting. Given the likely helpfulness of these first aid responses, they need greater promotion in the community.

Other first aid responses were mentioned only by a minority. Of particular concern is the low percentage assessing risk of harm for the person in the depression/suicidal vignette. Asking about suicidal intentions is often recommended as a response [13,14], although there is no evidence on whether this actually helps prevent suicide. Previous research has investigated first aid responses of young people to suicidal intent in their peers, finding that many would not tell a responsible adult about it [15]. However, we are unaware on any previous research on adults' responses to suicidal intent in someone they know.

Encouraging self-help was another minority response, but was associated with stigma and lack of recognition of the mental disorder in the vignette. Respondents appear to have suggested self-help as an alternative to professional help, rather than as a complement to it. We have previously reviewed the evidence on self-help interventions for depression and anxiety disorders and found that some have support [16,17]. Such interventions need to more widely promoted, but not as a substitute for professional help.

When correlates of first aid responses were examined, most variables had at least one significant association. However, the variables that most often predicted first aid responses were female gender, low personal stigma and correct recognition of the disorder in the vignette. The latter two predictors indicate potential barriers to providing first aid. Respondents who saw the person in the vignette as having negative attributes were less likely to respond by encouraging professional help-seeking or providing personal support. Efforts to reduce stigma in the community may therefore facilitate greater first aid. People who did not recognize the disorder showed a similar pattern of responses. These people lack knowledge of mental disorders, at least to the extent of being able to apply a psychiatric label. Therefore community education about how to recognize these disorders may also facilitate helpful first aid responses.

One approach to improving public responses to people with a mental disorder is an individual training course in mental health first aid [18]. Such a course has been developed and teaches an action plan with five steps of mental health first aid: (1) Assess the risk of suicide or harm; (2) Listen non- judgementally; (3) Give information and encouragement; (4) Encourage person to get appropriate professional help; and (5) Encourage self-help strategies. [19]. Two randomised controlled trials of this course, one in a work place environment [20] and the other with members of the public in a rural area [8], have shown benefits of the training: better recognition of disorders, changes in beliefs about treatment to be more like those of professionals, decreased social distance, increased confidence in providing help, and increase in actual help provided. The workplace trial also found improved mental health in course participants [20].

The study has two limitations which must be acknowledged. The major one is that the study has assessed intended first aid to a hypothetical person in a case vignette. Whether these intentions would be implemented in practice is unknown. Intentions might be seen as placing an upper limit on responses, such that if a respondent fails to state an intention, this is unlikely to be seen in practice. An alternative approach would have been to ask the respondent how they had treated actual people they knew with mental health problems. However, the disadvantage of this alternative would have been the lack of standard situations. A second limitation is that the inter-rater reliability of coding some of the first aid responses was low. Conclusions about these responses must be viewed with caution. On the other hand, the strengths of the study are the large representative sample, the open-ended responses which did not constrain the respondents to particular alternatives, and the ability to compare responses to a series of standard scenarios.

## Conclusions

There is room for improving the range of mental health first aid responses in the community. Lack of knowledge of mental health and stigmatizing attitudes are important barriers to effective first aid.

## Competing interests

The author(s) declare that they have no competing interests.

## Authors' contributions

AFJ was involved in securing funding for the survey, had a major role in the design of the survey and the interview questionnaire, carried out the descriptive statistical analysis and had a major role in writing the manuscript.

KAB provided research assistance with the survey, coded all the responses, and wrote the method relevant to this coding.

KMG was involved in the design of the study, developed the stigma scales and wrote some of the manuscript.

BAK developed the coding scheme, was the second rater for inter-rater reliability, and wrote some of the manuscript.

RAP did the regression analyses and wrote the section of the Method describing this.

All authors read and approved the final manuscript.

## Pre-publication history

The pre-publication history for this paper can be accessed here:


